# Home advantage and mispricing in indoor sports’ ghost games: the case of European basketball

**DOI:** 10.1007/s10479-022-04950-7

**Published:** 2022-09-28

**Authors:** Luca De Angelis, J. James Reade

**Affiliations:** 1 Department of Economics, University of Bologna, Bologna, Italy; 2 Department of Economics, University of Reading, Reading, England

**Keywords:** Sports forecasting, Market efficiency, Home advantage, Betting markets, COVID-19

## Abstract

Several recent studies suggest that the home advantage, that is, the benefit competitors accrue from performing in familiar surroundings, was—at least temporarily—reduced in games played without spectators due to the COVID-19 Pandemic. These games played without fans during the Pandemic have been dubbed ‘ghost games’. However, the majority of the research to date focus on soccer and no contributions have been provided for indoor sports, where the effect of the support of the fans might have a stronger impact than in outdoor arenas. In this paper, we try to fill this gap by investigating the effect of ghost games in basketball with a special focus on the possible reduction of the home advantage due to the absence of spectators inside the arena. In particular, we test (i) for the reduction of the home advantage in basketball, (ii) whether such reduction tends to disappear over time, (iii) if the bookmakers promptly adapt to such structural change or whether mispricing was created on the betting market. The results from a large data set covering all seasons since 2004 for the ten most popular and followed basketball leagues in Europe show, on the one hand, an overall significant reduction of the home advantage of around 5% and no evidence that suggests that this effect has been reduced at as teams became more accustomed to playing without fans; on the other hand, bookmakers appear to have anticipated such effect and priced home win in basketball matches accordingly, thus avoiding creating mispricing on betting markets.

## Introduction

The Covid-19 pandemic has dramatically impacted professional sport. Following an initial almost complete suspension of sporting competitions around the world, professional sports resumed competitions, but in most cases this has been either without fan attendance, or with restricted attendances, at sporting venues. As has been shown in many studies, fan attendance is an influential factor in determining the final outcome of a game.

It is commonly assumed that individuals will perform better when there is a crowd supporting them (Schwartz & Barsky, 1977). Crowd support is considered one of the most decisive factors of home bias or home advantage, the tendency for sporting teams to perform better at their home ground than away from home. It is argued that crowd support, in addition to encouraging the home team, discourages opponents and psychologically influences the behaviour of the referees in favour of the home team (Nevill et al., 2002).^1^ Home bias is one of the most documented phenomena across all sports. In their influential review, Courneya and Carron (1992, p.13) defined the home advantage in team games as “the consistent finding that home teams in sports competitions win over 50% of the games played under a balanced home and away schedule". In unbalanced schedules such as cup competitions, it is common to consider home advantage in relation to the relative strengths of the two teams involved. A home advantage exists if a home team wins more often than the relative quality difference betwen the teams in matches suggests they should.

The extraordinary situation caused by the pandemic provided an unprecedented natural experiment, allowing us to analyse an extended period of consecutive ghost games. The analysis of the impact of ghost games in soccer competitions has been provided by a number of contributions in recent literature, such as Fischer and Haucap (2020), Meier et al. (2020) and Dilger and Vischer (2020). However, to the best of our knowledge, no contribution exists on the impact of ghost games for indoor sports, that is where the effect of the support of the fans might have a stronger impact than in big stadia, as Schwartz and Barsky (1977) argued.

In this paper we evaluate the impact of ghost games in basketball by examining the reduction of the home advantage bias due to the absence of supporters using the current top ten European basketball leagues.^2^ In particular, we first investigate and quantify the home advantage bias in European league basketball and test for a possible impact of the ghost games on this advantage, evaluating whether such impact is somewhat temporary or permanent. The impact may be temporary if the home advantage is caused by familiarity, since home teams may be unfamiliar with playing in their stadiums but without fans. As they adapt to their home stadium without fans, it might be expected that the home advantage returns, and the reduction from Covid-19 is temporary. Moreover, we aim at evaluating the efficiency of online betting markets for the European leagues. In particular, we investigate whether betting markets are (weak-form) efficient and whether and how they adapted to the structural break of ghost games.

In this paper we address the following list of research questions:

**1a.**: Does the home advantage exist in basketball?
**1b.**: Is there an impact of ghost games on the home advantage?
**1c.**: Is this effect persistent over time?
**2a.**: Are bookmakers efficient in pricing basketball matches?
**2b.**: Have they adapted their prices to consider the possible effect of ghost games?

Our whole sample consists of 27,691 matches from 2004 to 2021, of which 1,026 are ghost games. Overall, the home win percentage before Covid-19 pandemic, that is when ghost games were just exceptions, was about 61%. Following the closure of the arenas the percentage of home winning decreased to about 56%.

Moreover, we do not find evidence of market inefficiency neither before nor after the introduction of the ghost games due to the outbreak of the Covid-19 pandemic. In particular, we find evidence that bookmakers have anticipated the effect of ghost games in their forecasts.

The paper is structured as follows. Section 2 discusses the relevant literature, Section 3 outlines the methodology, Section 4 shows the results of the empirical analysis, and Section 5 concludes.

## Literature review

### Home advantage

Home advantage, defined as the benefit that the home team is said to gain over the visiting team simply from playing on its own home field, is a topic that has attracted the attention of many studies, starting with the seminal work by Schwartz and Barsky (1977), who found that this bias has existed in selected American team sports for a long period of time. In particular, they found that the advantage of playing at home field differs from one sport to another, with greater advantage within indoor sports, such as basketball and ice hockey, relative to the outdoor sports, such as baseball and football. Moreover, they identified the three main sources of this bias: learning factors, travel (fatigue) factors and crowd factors. Over time, researchers have investigated specific aspects of home advantage, focusing on different issues, such as the psychological sphere of the players, the behaviour of spectators, the conditioning of the referees or the type of travel to the different locations.

From a psychological perspective away teams may be regarded as a sort of “invaders”, causing home team players to energize additional resistance forces, as measured by higher testosterone levels in home players (Neave & Wolfson, 2003; Carré et al., 2006). Relatedly, home team players show a higher self-esteem and self-efficiency (Terry et al., 1998; Waters & Lovell, 2002), that can be interpreted as higher self-confidence and determination, and lower fear of the game when they play on the home field (Bray et al., 2002).

The sources of home advantage have been—and likely will always be—a matter of debate. While every study agrees on the existence of this phenomenon, *“... the exact source of the home advantage is impossible to pinpoint from the inherently ambiguous archival data that home advantage research typically rely upon”* (Wallace et al., 2005, p.429). Home advantage in soccer appears to be linked to the crowds turnout, suggesting that home advantage is more relevant in divisions with larger crowds (Nevill et al., 1996). This is in line with earlier studies showing that the magnitude of home advantage significantly increases in crowd density (Courneya & Carron, 1992; Agnew & Carron, 1994). Evidence of home advantage was found also in basketball, where the crowd factors appeared to be its most relevant determinants (Nevill & Holder, 1999). Moreover, the effect of home advantage in the professional leagues of France, Greece, Italy and Spain was found to be higher than in NBA (National Basketball Association) (Pollard & Gómez, 2007).

While the earlier studies focused on audience behaviour and what it entailed, in recent years, and especially now in the wake of the pandemic, the focus has shifted to analysing the consequence of the absence of the public. Indeed, while crowd support is likely to be a major factor, the precise way in which it produces a home advantage has yet to be pinpointed (Pollard & Pollard, 2005). Accordingly, it has been noted that spectators may directly influence competitive outcomes by affecting player performances (Scoppa, 2008; Sanders & Walia, 2012). Crowds know their crucial role in supporting their team: fans in soccer crowds believe that they can indeed affect the outcome of a match in their own team’s favour, by influencing the referee’s decisions (Wolfson et al., 2005). Through an investigation of the impact of social pressure on the outcomes of historical European soccer matches, Reade et al. (2020) found that the substantial commonly observed home advantage was disproportionately eroded when fans were absent.

The behavior of referees is another determining factor for home bias, closely linked to the crowd factor (Schwartz & Barsky, 1977). Indeed, there is evidence that the noise of a home crowd is a cause of referee bias (Pollard & Pollard, 2005). Researchers have also focused on how referees react to home crowd pressure. Large and statistically significant effects on the number of yellow cards issued by referees were found, with fewer cards were awarded to the away teams in absence of a crowd (Bryson et al., 2021). In contrast, other studies suggested that rather than penalising the away players more, the dominant effect of crowd noise was to push qualified referees to penalise the home players less (Nevill et al., 2002). In any case, the unintentional reaction to positive and negative reinforcement undertaken by the home crowd suggests that referees are, on average, partial to home team in taking discretionary decision (Sutter & Kocher, 2004). This referee bias is reflected in some statistics including numbers of fouls, disciplinary sanctions and additional time awarded. Garicano et al. (2005) identified a systematic bias among referees in the top levels of Spanish soccer. They found that referees systematically shortened matches when the home team was winning and lengthened them when the home team was losing. Rocha et al. (2013) showed that this source of referees bias is more prevalent in Brazilian soccer when matches are televised. Moreover, there is a correlation between card difference, given for violations, and chances of winning (Frondel & Schubert, 2016). It has also been argued that better referee training over time is one of the reasons for the declining home advantage over recent years (Nevill et al., 2013).

Finally, travel is another factor affecting home bias. However, the literature has found contradicting results, possibly due to the ever increasing professionalization of the teams and the rising convenience of traveling. On the one hand, Lambert and Du Preez (2007) found that travel did not contribute to home advantage. On the other hand, other studies suggest that travel has a small but significant role in home advantage (Entine & Small, 2008). In European basketball during the Covid-19 Pandemic, teams continued to travel for contests within their leagues, and although undoubtedly with various lockdowns travel was more costly than would ordinarily be the case, we are nonetheless unable to identify any travel effect with Covid-19 related modifications to competitions in basketball.

### Market efficiency

A well-established framework for discussing market efficiency is provided by the renowned Efficient-Market Hypothesis (EMH), according to which market efficiency can be categorized into a weak, semi-strong or strong form depending on the amount of information reflected in prices (Malkiel & Fama, 1970). In the weak form, the current prices reflect all the information that is contained in historical prices, thus ruling out the possibility of achieving excess returns using an analysis of past prices alone. In the semi-strong form, efficiency market prices reflect not only the information contained in the historical price series, but also any other public information; therefore, it is not possible to formulate a trading strategy with an expected return higher than the market return on the basis of public information alone. Lastly, in its strong form the efficiency occurs when market prices reflect, in addition to the above, any private information (that is, all the information); there is no way to forecast to make profit.

Given its importance for investment opportunities and investor roles, the question of market efficiency has attracted abundant empirical research. The concept of market efficiency is applicable to many types of markets, from traditional stock markets to betting markets. The latter, not unlike traditional markets, are characterised by a large number of experienced investors (bettors) with access to information and assets (betting contracts) acting in the market. The higher concentration of educated investors though in betting markets make them an ideal setting to study market efficiency (Hvattum, 2013). Indeed, it can be argued that betting markets are better suited to testing market efficiency and rational expectations than stock or other asset markets (Thaler & Ziemba, 1988). This is because in betting markets each asset or bet has a well-defined termination point at which its value becomes certain, and its outcome is not affected by macroeconomic factors or bettor expectations (Flepp et al., 2017).

Most of the studies on information market efficiency focuses on the weak-form of information market efficiency. For instance, market inefficiencies as deviation from the weak form market efficiency were found in English soccer (Dixon & Coles, 1997; Rue & Salvesen, 2000; Kuypers, 2000; Dixon & Pope, 2004). The deviations from the weak form market efficiency for European soccer betting market may well be the result of differences across bookmakers and players, variation in information and products, and behavioural biases of punters (Vlastakis et al., 2009). Goddard and Asimakopoulos (2004) found evidence of generating positive returns when betting at the end of the season games, while Marshall (2009) and Brown et al. (2019) noted that markets need some minutes to converge to an efficient level when arbitrage opportunities arise between different market participants. Temporal market inefficiencies were also found when betting on recently promoted teams, as the change of league is often accompanied by many changes in a team’s roster which complicate predictions about such teams (Deutscher et al., 2018). Moreover, a weak-form market efficiency in the case of the European soccer major leagues was identified using a forecast-based approach (Angelini & De Angelis, 2019; Elaad et al., 2020).

While the weak form certainly plays a predominant role in the literature on the efficiency of betting markets, studies on the semi-strong form can also be found. A semi-strong efficient market requires prices to immediately reflect new information once it becomes public knowledge. Indeed, evidence from sports betting exchanges shows that prices update swiftly following a scored goal in soccer, suggesting that betting markets seem to incorporate market news rapidly and completely (Croxson & Reade, 2014). Conversely, Choi and Hui (2014) rejected the hypothesis of semi-strong market efficiency: using similar live soccer betting data, they found that prices generally underreact to normal news and overreact to surprising news. Semi-strong market inefficiencies are also detected by Angelini et al. (2022). With respect to tennis, examining court-side trading during live matches, Bizzozero et al. (2018) suggested that the fast traders promote quick price discovery and correctly incorporate new information into prices.

Previous studies have mainly focused on behavioral biases such as: (i) the favourite-longshot bias; (ii) the sentiment bias; (iii) the mispricing of the home advantage. The favourite-longshot bias has attracted much attention. This type of bias is encountered when favourites win more often than the subjective market probabilities imply, and longshots less often. Various theories exist to explain the existence of this bias, which is perceived as an important deviation from the market efficiency hypothesis. The main theories proposed by the literature are reviewed by Ottaviani and Sørensen (2008), who argue that bettors who are willing to take risks accept a lower expected payout when betting on longshots. Unlike fixed-odds bookmaker betting markets, the presence of a reverse favourite-longshot bias was suggested by Angelini et al. (2022), who tested the weak form market efficiency by analysing pre-match exchange odds, and the semi-strong form efficiency by focusing on the in-play odds after the arrival of the major news that the first goal of a soccer match had been scored. Both in-play and pre-match exchange odds revealed a reverse favourite-longshot bias that could have been exploited to make profits. The sentiment bias arises when bettors place their bets for reasons that do not reflect technical or fundamental factors, e.g. team popularity, affecting the likelihood of a team winning. Notorious examples of bettor’s sentiment include the optimistic perception bias, which causes bettors to overrate the winning probability of certain teams (Kuypers, 2000; Levitt, 2004; Page, 2009), and the loyalty bias (Forrest et al., 2005; Franck et al., 2011), which prevents bettors from betting against the team they support. Finally, there is evidence of a persistent mispricing of the home advantage in betting odds in several betting markets. For example, bias in the pricing of the home field advantage in point spread betting market has been observed in American football and more specifically in the NFL. In particular, it was found that bettors appear to misprice the home field advantage in game with national focus (Monday night and playoff games), and that home team underdogs win at a rate sufficient to reject both the unbiased forecast and absence of profit opportunities version of efficiency (Vergin & Sosik, 1999). Evidence of mispricing of the home field advantage has also been found in basketball and baseball in either regular season or playoff games (Gandar et al., 2001).

### European basketball

Despite the popularity of basketball in Europe—the sport is second only to soccer in almost all of the European countries—the literature devoted to this sport in Europe is relatively small. Econometric and OR approaches have been proposed for overseas basketball leagues such as the NBA (Yang et al., 2014; Moreno & Lozano, 2014; Cervone et al., 2016; Xin et al., 2017; Sandholtz & Bornn, 2020) and the Argentinean league (Durán et al., 2021). However, the contributions dealing with European basketball are rather limited. The importance of quantitative analyses to help in supporting the decision making process of any coach both before and during an European basketball game has been stressed by Nikolaidis (2015). For instance, Facchinetti et al. (2021) use data from GPS sensors to analyse the on-field performance of single players and the whole team in three games of the Italian Basketball Cup Final Eight 2017.

Also when considering betting markets, the existing literature is mainly devoted to the NBA (Paul & Weinbach, 2008; Hubáček et al., 2019) and, to the best of our knowledge, this paper is the first to investigate the fixed-odds bookmaker’s markets for the top ten basketball leagues in Europe. Our analysis extends the one developed by Angelini and De Angelis (2019) for soccer betting markets to the case of basketball and to evaluate the potential adaptation of bookmakers to the structural break provided by the Covid-19 induced ghost games.

### Home advantage and market efficiency during Covid-19

In this section, we provide an overview of recent studies on home bias and the efficiency of sports betting markets which exploit the increased frequency of ghost games brought about by the pandemic. As this literature focuses on soccer, to the best of our knowledge we are the first to study the effects of Covid-19 induced ghost games on home advantage and on the efficiency of betting markets in basketball.

The effect of ghost games on home advantage due to the pandemic does not appear to be uniform. For example, a decrease of the home advantage following an increase in ghost games was documented in the first division of German soccer, while it did not occur for the lower second division league (Fischer & Haucap, 2020). This can be partially explained by the relative importance of the first division clubs and the higher turnout of fans in normal times due to larger stadiums, which might make first division clubs more responsive to the lack of support (Fischer & Haucap, 2020). Teams in the top German league, the *Bundesliga*, experienced a decrease in home team goals and an increase in away team goals during the ghost games induced by Covid-19 compared to earlier seasons (Winkelmann et al., 2021). This reduction in home advantage was found to be driven also by the complete disappearance of the referees’ home bias (Dilger & Vischer, 2020). Consistently with this finding, Wunderlich et al. (2021) analysed a much larger data set from several European leagues and found that increased sanctioning of away teams disappears in the absence of spectators, confirming the existence of crowd-induced referee-bias in standard times. Moreover, while the match dominance of home teams decreased significantly as indicated by shots, surprisingly only a non-significant decrease in home advantage was found.

Available studies on the efficiency of betting markets following Covid-19 have focused not only on static and retrospective perspectives on market efficiency, but also on the adaptation process of match-related expectations due to new experiences. This can be analysed either from the point of view of a single match, that is in-play betting markets (see, among others, 2022), or by observing the response of these markets to unforeseen structural changes, as is the case of ghost games in the major European soccer leagues during the Covid-19 pandemic (Meier et al., 2020; Fischer & Haucap, 2020; Dilger & Vischer, 2020). Bookmakers did not accurately predict the Covid-19 induced ghost games and their impact on the home and away teams’ winning probabilities, suggesting an inefficiency of markets at least in semi-strong form: indeed, bookmakers systematically overestimate (underestimate) the home (away) teams’ winning probability during the early stage of post resumption period (Meier et al., 2020). Analyzing the two major German soccer league before and after the interruption of championships due to Covid-19, Fischer and Haucap (2020) found that betting markets expected similar small reductions in the home advantage in the two main professional soccer divisions, and that the very different match outcomes between the two leagues over the course of the ghost game season did not result in a proper adaption of expectations, pointing at inefficiencies in the market. A bookmakers’ mispricing was also found in the German league, where the bookmakers’ odds did not reflect the reduction in home advantage, thus determining a possible profit strategy betting on away teams that would generate a gain of almost 15% (Dilger & Vischer, 2020).

## Methodology

In this section we briefly outline the methodology used to address the research questions posed in the introduction.

### The impact of ghost games on the home advantage in European basketball

To answer research questions **1a-1c** we first implement a linear probability model where the dependent variable, $y_i$, is a dichotomous variable that captures the home win. Hence, $y_i=1$ in the case of the home team winning and $y_i=0$ otherwise (away team win). As regressor of the linear probability model we define the main variable of interest in our analysis that is the dummy related to the Covid-19 induced ghost games (labelled GG), where $GG_i=1$ if the match is played without fans and 0 otherwise. Moreover, we control for playoff games, league effects and, to evaluate if there are either temporary or permanent effects, also a count variable which denotes the number of ghost games played by a specific team. The rationale behind the inclusion of the playoffs variable in the model specification can be explained by recalling that, after the (round-robin) regular season, only the top eight teams are allowed to compete for the title in a three round best-of series competition where the teams which ranked top at the end of the regular season have the chance to play at home the majority of the playoff games. For instance, in the first round of the playoffs the team that ranked first at the end of the regular season plays against the team that ranked eighth in a best of three games to advance to the next stage (i.e. the semifinals). Therefore, since the playoffs’ team pairing is not random and the teams that play more games at home are theoretically the more likely to win, especially in the first round, we expect that the home advantage is stronger in the playoff games than during the regular season.

The general model we consider is thus the following:

1 $$\begin{aligned} y_i= & {} \beta _0 + \beta _1 GG_i + \beta _2 Playoff_i + \beta _{3} Matchday_i + \beta _{4} Matchday_i^2\nonumber \\&+ \beta _{5} \mathbbm {1}(League_{i}= j) + \beta _{6} [GG_i \cdot \mathbbm {1}(League_{i}= j)] + u_i \end{aligned}$$

where, for $$i = 1,\ldots ,N$$, $$y_i$$ denotes a dummy variable for home win, $$GG_i$$ is the ghost games dummy, $$ Playoff_i=1$$ if the match is a playoff or final phase game and 0 otherwise (regular season matches), $$ Matchday_i$$ is a count variable which denotes the number of ghost games played by team *i*, e.g. $$ Matchday_i =3$$ implies that team *i* plays its third game at home behind closed doors and has already played two ghost home games, and $$\mathbbm {1}(League_{i}= j)$$ denotes an indicator function for the condition that team *i* belongs to league *j*, for $$j=1,\ldots ,J$$. In model (1), a rejection of the null hypothesis $$H_0: \beta _1=0$$ in favour of the alternative hypothesis $$H_1: \beta _1<0$$ (one-sided *t*-test) can be interpreted as a statistically significant reduction of the home advantage due to the lack of fans’ support inside the arena induced by ghost games. A significant value of $${\widehat{\beta }}_3$$ (but not $${\widehat{\beta }}_4$$) would entail a linear adjustment of the home advantage during the ghost games period. If also $${\widehat{\beta }}_4$$ is found significantly different from zero, then this adjustment would be nonlinear. Assuming the presence of a reduction of the home advantage during ghost games (i.e. finding evidence of a significant negative value of $${\widehat{\beta }}_1$$), the adjustment provided by the quadratic form for the Matchday variable could be either permanent, i.e. the home advantage returns to its pre-Covid levels, or transitory, i.e. the reduction in home advantage is not fully absorbed even after the teams have played several games without the support of their fans. Moreover, while coefficient $$\beta _5$$ captures the different average probability of home team winning in different leagues, a rejection of the null $$H_0: \beta _6=0$$ would entail a significant specific ghost game league effect. The main advantage of the linear probability model is the ease of interpretation of the estimated coefficients. However, there are well-known issues with the functional form as the predicted probabilities, $$ P(y_i=1 | X_i) $$, where $$X_i$$ denotes the vector of regressors, may be greater than one or smaller than zero. Although non-linear models such as logit and probit are more appropriate when modelling dichotomous dependent variables, the issue is mainly related to the extreme (predicted) values of the cdf, i.e. when $${\widehat{y}}_i$$ is either close to 0 or 1. This is not the case in our application, because the home win probability takes values around 0.6, as it can be noted from the results reported in Section 4. As a matter of fact, in our case, the results achieved using logit and probit models are almost identical to the ones obtained with the linear probability models.^3^ Since the estimation is done with sparse dummy variables (recall, e.g., that variable GG represents only 3.7% of the sample size), the standard White’s heteroskedasticity-robust covariance matrix estimators could be quite imprecise. Following Hansen (2021), a possible solution is to replace the standard biased covariance matrix estimator with the conservative estimator $${\widehat{V}}^{HC3}_{{\widehat{\beta }}}$$, using the squared prediction errors instead of the squared residuals. Therefore, we adopt a HC3 conservative standard error estimator throughout the empirical analyses in Section 4.

Note also that the model in (1) is akin to the difference-in-difference approach. In particular, we test for the ‘treatment’ effect (i.e. the effect of the absence of fans) on the home advantage using the post-Covid (ghost game) sample as the ‘treated’ group and the pre-Covid sample as the ‘control’ group.

### Efficiency of online European basketball betting markets

To answer research questions **2a** and **2b** related to the unbiasedness of the predictions made by bookmakers, we use the Mincer-Zarnowitz forecasting regression-type analysis as used by Angelini and De Angelis (2019) to test for efficiency in betting markets. In this framework, we test whether the bookmaker’s forecasts of the (implied) probability that a home team will win are optimal.^4^ In particular, the optimality property is achieved when the bookmaker’s forecast errors is orthogonal to any regressors that belong to the information set available when the ex-ante forecast has been made. A straightforward way to test the optimality property is to regress the forecast error on a constant and regressors that belong to the information set, e.g. the implied probability itself and interactions of this forecast with other variables as, for instance, the ghost games dummy, and jointly test that all the coefficients are not significantly different from zero.

In particular, as shown in Angelini and De Angelis (2019), let $y_i$ be distributed as a Bernoulli with (true) probability $\pi _i$. Assuming $\Omega _i$ to be the hypothetical information set that contains all the information in the universe, then $y_i | \Omega _i \sim Bin(1, \pi _i)$. Moreover, let $o_i$ be the odds for a particular outcome of the match $i$ (in our case, the home win), and $p_i$ be the corresponding implied probability forecast, where $p_i = 1 / o_i$. The bookmaker’s unbiased forecast is given by $\tilde{p_i} = E (y_i|{\mathscr {F}}_i)$, where ${\mathscr {F}}_i$ is the information set available to the bookmakers on match $i$ and it is a subset of the full information set ${\mathscr {F}}_i \subset \Omega _i$. Since the bookmakers are profit-oriented agents, their primary source of income is coming from the commissions (i.e. the bookmaker’s margin). The margin, also called the *vig*, is a “fee” charged by the bookmaker that is reflected in the odds offered to the bettor in order to ensure a profit regardless of the outcome. In particular, the bookmaker’s margin, which we will denote by $\kappa _i$, is such that the odds offered to the bettors are lower than the actual probability of a outcome occurring, making the sum of the *implied* probabilities of the different possible outcomes greater than 1. The bookmakers’ probability forecast that is *de facto* employed to set the odds offered in the market is therefore given by

$$\begin{aligned} {p}_i = E(y_i | {\mathscr {F}}_i) + \kappa _i \text{ with } \kappa _i>0. \end{aligned}$$

The bookmaker’s margin $\kappa _i$ is generally not fixed and can change between games, bookmakers and over time. A possible popular solution to circumvent this problem is to normalise the odds, that is to divide the inverse odds by the sum of the inverse odds:

2 $$\begin{aligned} p_{i,j} = \frac{1/o_{i,j}}{1/o_{i,j}+1/o_{i,j'}} \end{aligned}$$

where $o_{i,j}$ and $o_{i,j'}$ denotes the odds for the home win and away win, respectively. The results for the normalised odds are shown in the Appendix. The bookmaker’s forecast error for the outcome of match $i$ is $\epsilon _i = y_i - {p}_i$ and, under the null hypothesis of market efficiency, $\epsilon _i$ should be zero. However, since $p_i$ overstates the true probability $\pi _i$ (that is, $p_i > E ( y_i | \Omega _i)$ because of margins $\kappa _i$), the conditional expectation of $\epsilon _i$ is equal to minus the bookmakers’ average margin, i.e. $E (\epsilon _i | {\mathscr {F}}_i) = - \kappa$; see Angelini and De Angelis (2019) for more details. The market efficient hypothesis can thus be tested by estimating the following model (either for the whole sample or for individual leagues):

3 $$\begin{aligned} \epsilon _i = \alpha + \beta p_i + \upsilon _i, \ \ \text{ with } \upsilon _i \sim \text{ i.i.d. } (0, \sigma ^2_i) \ \ \ i = 1,\ldots , N \end{aligned}$$

where *N* is the number of matches and the constant $\alpha$ captures (minus) the bookmakers’ average margin. The coefficient of interest is $\beta$, which represents the effect of the implied probabilities $p_i$ on the forecast error, and by analysing its statistical significance we can infer the unbiasedness of the market. Indeed, market efficiency would imply that the conditional expectation $E(\epsilon _i | {\mathscr {F}}_i)= \alpha$, such that a rejection of the null hypothesis $H_0: \beta = 0$ would imply that the market is not unbiased.

Although Eq. (3) is sufficient to identify biases on the market and possible price (odds) distortions due to, e.g., bettors’ bias exploitation, we want to shed further light on the possible sources of inefficiency in the betting market as a result of ghost games and the consequent decrease in home advantage. To do this, a number of regressors related to ghost games are added to the specification of the basic model (3). More specifically, we consider the following regression model:

4 $$\begin{aligned} \epsilon _i = \alpha + \beta _{1}p_i + \beta _{2}GG_i + \beta _{3}FirstGG_i + \beta _{4}(GG_i \cdot p_i) + \beta _{5} (FirstGG_i \cdot p_i) + \upsilon _i\nonumber \\ \end{aligned}$$

where $FirstGG_i$ is a dummy variable with value of 1 if the match is among the first 3 matches played by home team $i$ without the presence of the fans, to assess whether there is a short-term temporal impact of ghost games. Interactions between $GG_i$ and $p_i$ and between $FirstGG_i$ and $p_i$ are also included to evaluate whether there is a significant marginal impact on the forecast error of the implied probabilities for all the ghost games or just the first three games played behind closed doors. Ioannidis and Peel (2005) showed that forecast errors can exhibit heteroskedasticity under the null of market efficiency. Therefore, the estimates of the regressions (3) and (4) are obtained through Weighted Least Squares (WLS), where the $N \times N$ diagonal matrix with elements $\sigma ^2_1,\ldots , \sigma ^2_N$ is used as weights. In this setup, $\sigma ^2_i$ in Eq. (3) can be approximated by the variance of a Bernouilli variable, i.e. $\sigma ^2_i=p_i (1 - p_i)$.

Moreover, to evaluate the degree of market unbiasedness and whether any biases are large enough to provide profitable opportunities for bettors, which in turn would imply market inefficiency, in line with Angelini and De Angelis (2019), we derive the “efficiency curve” considering the fitted values from the estimation of the models in Eq. (3) for all possible probability values:

5 $$\begin{aligned} {\widehat{G}}(p_G)={\widehat{\alpha }}+{\widehat{\beta }}p_G, \ \ p_G \in (0,1) \end{aligned}$$

where ${\widehat{\alpha }}$ and ${\widehat{\beta }}$ are the estimates of the parameters in Eq. (3). The related confidence bands are computed as:

$$\begin{aligned} CI_J = [{\widehat{G}}(p_G)-z_{\alpha /2}s.e.({\widehat{G}}(p_G)), {\widehat{G}}(p_G)+z_{\alpha /2}s.e.({\widehat{G}}(p_G))] \end{aligned}$$

where $s.e.({\widehat{G}}(p_G))=[\bigtriangledown {\widehat{G}}(p_G)' V_{WLS} \bigtriangledown {\widehat{G}}(p_G)]^{(1/2)},$ $z_{\alpha /2}$ is the $100(1-\alpha /2)$th percentile of the standard normal distribution, $\bigtriangledown {\widehat{G}}(p_G) = (1, p_G)'$ is the gradient and $V_{WLS}$ is the variance of the WLS estimator. If we fix a value for $p_G$, i.e. $p_G^0 \in (0, 1)$, then ${\widehat{G}}(p_G^0) = 0$ implies market unbiasedness. Conversely, when ${\widehat{G}}(p_G^0) \ne 0$ we find evidence of bias, and the sign of ${\widehat{G}}(p_G^0)$ indicates which of the two sides, i.e. the bettors or the bookmakers, might profit from this bias. Basically, when ${\widehat{G}}(p_G^0)$ is greater than 0, the inefficiency is due to the fact that bettors might profit from it, whereas ${\widehat{G}}(p_G^0)$ is less than 0, would entail profits for bookmakers.

### Determinants of bookmakers’ odds

As further investigation of the bookmakers’ adaptation to the structural change provided by the introduction of the ghost games, we run simple linear regressions that consider the bookmaker’s (average) implied probability as dependent variable and, as regressors, the dummy variables for ghost games and playoffs, the matchday and its squares as well as a proxy of the strength of the home team, i.e. the Elo rating in its weighted version (WElo) as recently proposed by Angelini et al. (2022). Specifically, we evaluate the following general regression model:

6 $$\begin{aligned} p_i = \delta _0 + \delta _1 GG_i + \delta _2 Playoff_i + \delta _3 WElo_i + \delta _4 Matchday_i + \delta _5 Matchday^2_i + \xi _i\nonumber \\ \end{aligned}$$

With model (6) we aim at investigating biases in implied probabilities and whether the bookmaker’s odds have adapted, promptly or after a while, to the impact of ghost games on the home advantage. Note that the introduction of the (weigthed) Elo rating system in model (6) allows us to add a relevant proxy for the team’s strength and its likelihood to win the game against that specific opponent. Indeed, the Elo ratings is a method to estimate the strength of the teams based on the history of the matches played up to the match before the one under consideration. The most important difference between the classic Elo and the WElo proposed by Angelini et al. (2022) is that the latter does not only take into account the history of wins and losses but also the score with which these past matches ended, and thus how the victory or defeat was achieved. This weighted version provides more robust results than the standard Elo and more accurate predictions; see Angelini et al. (2022) for more details and both Elo and WElo ratings.

## Empirical analysis

### Data and preliminary analysis

The data are taken from *www.oddsportal.com*, a large database of comparative odds for numerous sports. We focus on the top ten basketball leagues according to the ranking updated at the end of 2020 in 15 European countries.^5^ More specifically, the leagues taken into account are the following: ACB Liga (Spain), VTB United League (Russia), Basketbol Süper Ligi (Turkey), LNB Pro A (France), Lega Basket Serie A (Italy), Basketball Bundesliga (Germany), HEBA Basket League (Greece), Adriatic League or ABA Liga (the participating countries have changed over time and now include Bosnia and Herzegovina, Croatia, Montenegro, North Macedonia, Serbia and Slovenia), Winner League (Israel), and the LKL (Lithuania). More details on each leagues, including the number of regular season home games, playoff structure, capacity of the arenas, details on the management of the coronavirus-affected 2019-20 season, and, where available, info on the attendances, are summarised in Table 8 in the Appendix.

The sample period varies from league to league depending on data availability, covering a time span that in its broadest form runs from 2004 until early 2021, for a total of 27,691 matches, of which 1,026 are ghost games.^6^ The data also comprise the odds offered by 47 international online bookmakers.^7^ Table 1 summarises the sample sizes considered in the different leagues and in the whole sample, broken down into matches with fans and ghost games.

**Table 1:** Composition of the data set

League	Country	Total	Pre-Covid	Post-Covid	Matchday	Sample
		games	games	games		period
ACB Liga	Spain	3927	3776	161	9	2004–2021
VTB United League	Russia	1947	1859	88	8	2009–2021
Basketbol Süper Ligi	Turkey	3002	2872	130	9	2007–2021
LNB Pro A	France	3488	3430	58	6	2004-2021
Lega Basket Serie A	Italy	3283	3175	108	8	2005–2021
Basketball Bundesliga	Germany	3858	3765	93	6	2004–2021
HEBA Basket League	Greece	2264	2188	76	7	2005–2021
ABA Liga	Adriatic League	2054	1960	94	8	2008–2021
Winner League	Israel	2182	2049	133	13	2008–2021
LKL	Lithuania	1686	1601	85	10	2011–2021
Total sample		27,691	26,675	1026		2004–2021

Matchday denotes the maximum number of ghost games played by at least one team at home

A data cleaning operation was carried out, eliminating matches with incomplete odds or matches in which the sum of the implied probabilities of the different outcomes was either smaller than 1 (284 matches) or larger than 1.25 (8 matches), therefore eliminating, respectively, potential arbitrage opportunities and illiquid markets as well as possible mistakes in the data.

Table 2 shows the percentage of home team wins for each league, pre-Covid and post-Covid.

**Table 2:** Descriptive statistics on home advantage

League	% Home Team win				
	Country	Overall (%)	Pre-Covid (%)	Post-Covid (%)	$\Delta$ (%)
ACB Liga	Spain	62.2	62.4	54.0	8.3$$^{**}$$
VTB United League	Russia	57.0	57.1	53.4	3.7
Basketbol Süper Ligi	Turkey	59.5	59.6	57.7	1.9
LNB Pro A	France	61.0	61.0	60.3	0.7
Lega Basket Serie A	Italy	63.7	64.0	52.8	11.3$$^{***}$$
Basketball Bundesliga	Germany	59.5	59.7	52.7	7.0$^{*}$
HEBA Basket League	Greece	63.5	63.4	65.8	–2.4
ABA Liga	Adriatic League	65.4	65.9	56.4	9.5$$^{**}$$
Winner League	Israel	57.7	58.0	54.1	3.8
LKL	Lithuania	57.7	57.6	58.8	–1.2
Total sample		60.9	61.1	56.0	5.1$$^{***}$$

$$*$$, $$**$$, and $$*\!*\!*$$ denote that the difference is significant at 10%, 5%, and 1% levels, respectively

A first glance at the percentages for the home advantage highlights that there has been an average decrease in home wins due to the closure of the arenas. The average probability of winning at home among all leagues before the outbreak of Covid-19 was 61.1%, in line with previous studies on indoor sports, e.g. Nevill and Holder (1999) found a home winning probability in basketball of 64.4%, while Gómez and Pollard (2011) found a home winning probability that ranged from 56.13% to 65.10% in different European leagues. In our sample, we find the biggest home bias in the ABA Liga, with a probability of winning of 65.9%, while the lowest is in the VTB United League (Russia) with 57.1%.

**Fig. 1:**
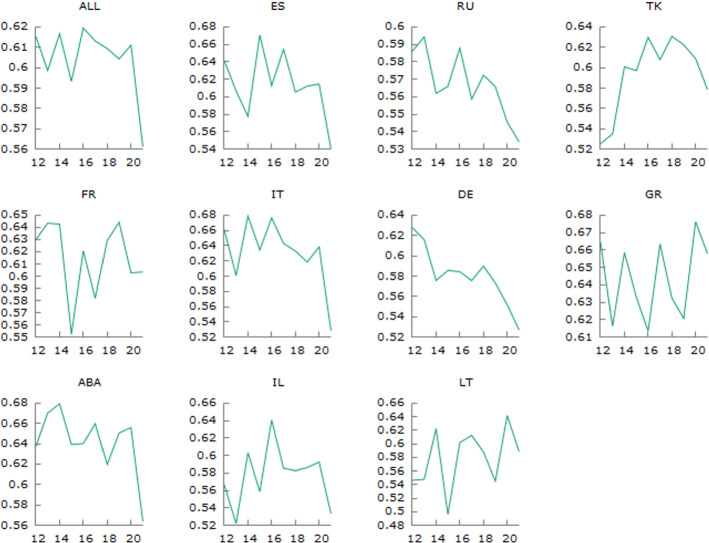
Realized home win probability for each league and season from 2011-2012 to 2020-2021

The results in Table 2 also show the decrease in home advantage, due to the absence of fans, in almost all the leagues taken into consideration, with the exceptions of the Greek and Lithuanian leagues, where we observe a small increase in the percentage of home wins. Excluding also the French league for which we do not observe a substantial change, all other leagues have experienced a concrete decrease in the percentage of home team victories, ranging from $-1.9\%$ (Turkey) to $-11.3\%$ (Italy). Performing a (one-sided) test on the percentages pre- and post-Covid outbreak, we reject the null hypothesis that such proportions are equal in the population for the Spanish, Italian, German and Adriatic leagues, as well as for the total sample. The abrupt drop in the home win advantage during the ghost game-affected 2020-2021 season is also evident from Fig. 1, especially for the leagues mentioned above.

Therefore, on a purely descriptive level, our first hypothesis of the existence of home advantage and the consequent decrease in the absence of an audience appears to be confirmed. In the next section we provide formal tests to evaluate our first three research questions, i.e. questions **1a-1c** in Sect. 1.

### Model results

In this section, we present the results from regression models and tests for the reduction of the home advantage during the ghost games period and the possible temporary effect of such reduction.

In Table 3 the results for the following linear probability models estimated by OLS using the whole sample are reported:

$$\begin{aligned} y_i= & {} \beta _0 + \beta _1 GG_i + u_i\\ y_i= & {} \beta _0 + \beta _1 GG_i + \beta _2 Playoff_i + u_i\\ y_i= & {} \beta _0 + \beta _1 GG_i + \beta _2 Playoff_i + \beta _3 Matchday_i + \beta _4 Matchday_i^2 + u_i \end{aligned}$$

where $$y_i$$ denotes a dummy variable for home win, $$GG_i$$ is the ghost games dummy, $$ Playoff_i=1$$ if the match is a playoff or final phase game and 0 otherwise (regular season matches), and $$ Matchday_i$$ is a count variable which denotes the number of ghost games played by team *i*. Note that in our sample the number of ghost games played by each team, and hence the values for Matchday variable, varies between leagues and ranges from 6 to 13 (see Table 1).

**Table 3:** Effect of ghost games on home wins

	Home win		
	(1)	(2)	(3)
GG	–0.0505***	–0.0474***	–0.1174**
	(0.0158)	(0.0158)	(0.0510)
Playoffs		0.0406***	0.0405***
		(0.0103)	(0.0103)
Matchday			0.0424*
			(0.0232)
Matchday$^2$			–0.0046**
			(0.0023)
const	0.6109***	0.6073***	0.6073***
	(0.0030)	(0.0031)	(0.0031)
Observations	27,691	27,691	27,691
Adj. $R^2$	0.0003	0.0009	0.0009
F-test (p-value)	0.0014	$<0.0001$	$<0.0001$

The dependent variable is all columns is an indicator for the home team winning. The model is estimated as LPM. *GG* is an indicator for whether the match had no fans. *Playoff* is an indicator for whether the match was a playoff match. *Matchday* is the number of times the home team has played behind closed doors in its own arena. Heteroskedasticity-robust standard errors (HC3) in parentheses.

$${^*}p<0.1$$, $${^{**}}p<0.05$$, $${^{***}}p<0.01$$

**Fig. 2:**
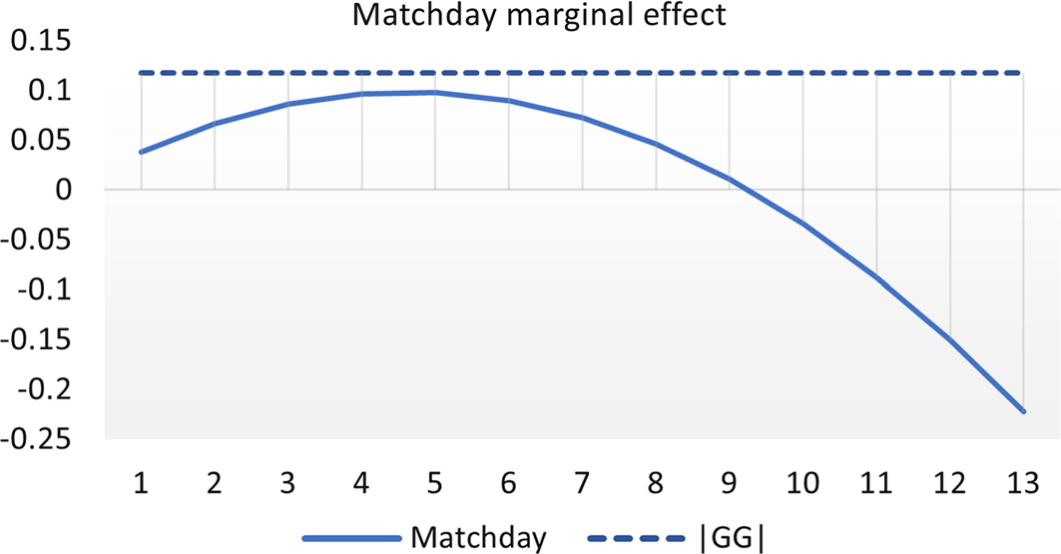
Marginal effect of Matchday variable on home advantage. |GG| denotes the estimated effect of ghost games (in absolute value)

The results in Table 3 show that the pre-Covid average proportion of home wins, which is captured by the constant of the models, is around 61%. Moreover, we find evidence that ghost games have a negative impact on the home winning probability, and significantly so for all the model specifications considered. Therefore, the absence of fans has the effect of significantly reducing home advantage, in our case by around 5% for models (1) and (2). As expected, playoff games have a significant positive effect on the probability of home win of about 4%.^8^ The results for model (3), i.e. in the case we include the nonlinear effect of the *Matchday* variable in the model specification, allow us to evaluate the transitory or permanent nature of the impact of ghost games in European basketball. In particular, to better assess the effect of the number of ghost (home) games played by the teams, we depict the marginal effect of the *Matchday* variable and (the absolute value of) the estimated home advantage reduction due to ghost game ($|{\widehat{\beta }}_1|$) in Fig. 2. From this figure, we note that the maximum value of the quadratic marginal effect is achieved for $Matchday \approx 5$, i.e. when the team has played five ghost games in its own arena. However, such maximum is not large enough to overcome the negative effect of ghost games, which in model specification (3) is estimated as ${\widehat{\beta }}_1=-0.1174$. Moreover, since this effect is concave (${\widehat{\beta }}_4<0$), there is no evidence that increasing the number of ghost games played by a team at home allows to restore the pre-Covid home advantage. This evidence suggests that the impact of the absence of fans in the arenas on the home advantage persists over time and it is not temporary as found in outdoor sports as soccer (Fischer & Haucap, 2020).

We then test whether the effects are different between leagues by estimating the following linear probability model:

$$\begin{aligned} y_{i} = \beta _0 + \beta _1 GG_{i} + \beta _2 Playoff_{i} + \beta _{5} \mathbbm {1}(League_{i}= j) + \beta _{6} [GG_i \cdot \mathbbm {1}(League_{i}= j)] + u_{i} \end{aligned}$$

The results are summarised in Table 4 and show that the reduction of the home advantage for individual leagues is not significantly different from the overall reduction as we do not reject the null hypothesis $H_0: \beta _6=0$. Therefore, we do not find evidence that the ghost game effect significantly vary among leagues and, albeit with different intensity, the absence of fans inside the arena creates a substantial reduction of the home advantage in all the top leagues in Europe. Conversely, it is interesting to note that the home winning probability significantly changes across leagues. As already noted from the descriptive statistics in Table 2, Russian VTB league shows the lowest probability of home winning (56.6%), while Balcanic ABA league shows the largest (65.7%). In summary, we observe league effects on the overall home advantage, i.e. different home winning probabilities across basketball leagues in Europe, but no differences among leagues are observed in post-Covid ghost game sample considered here.

**Table 4:** Effect of ghost games on home wins: league effects

	Home win				
	Constant	GG	Playoffs	League	GG $\cdot$ League
ACB Spain	0.6047***	–0.0412**	0.0412***	0.0178**	–0.0409
	(0.0034)	(0.0172)	(0.0103)	(0.0085)	(0.0438)
VTB Russia	0.6103***	–0.0480***	0.0414***	–0.0435***	0.0153
	(0.0032)	(0.0165)	(0.0103)	(0.0119)	(0.0574)
BSL Turkey	0.6091***	–0.0516***	0.0404***	–0.0161*	0.0355
	(0.0033)	(0.0169)	(0.0103)	(0.0097)	(0.0477)
LNB France	0.6073***	–0.0501***	0.0407***	0.0005	0.0456
	(0.0033)	(0.0163)	(0.0103)	(0.0089)	(0.0679)
Lega A Italy	0.6034***	–0.0399**	0.0394***	0.0322***	–0.0682
	(0.0033)	(0.0167)	(0.0103)	(0.0091)	(0.0520)
BBL Germany	0.6097***	–0.0465***	0.0404***	–0.0165*	–0.0199
	(0.0034)	(0.0166)	(0.0103)	(0.0086)	(0.0555)
HEBA Greece	0.6054***	–0.0533***	0.0401***	0.0240**	0.0818
	(0.0033)	(0.0165)	(0.0103)	(0.0108)	(0.0585)
ABA Adriatic	0.6031***	–0.0436***	0.0432***	0.0540***	–0.0497
	(0.0033)	(0.0166)	(0.0103)	(0.0112)	(0.0553)
ISR Israel	0.6099***	–0.0467***	0.0420***	–0.0354***	0.0094
	(0.0032)	(0.0169)	(0.0103)	(0.0113)	(0.0479)
LKL Lithuania	0.6096***	–0.0522***	0.0417***	–0.0389***	0.0698
	(0.0032)	(0.0165)	(0.0103)	(0.0127)	(0.0578)

The model is estimated as LPM. Heteroskedasticity-robust standard errors (HC3) in parentheses. $$^*p<0.1$$, $$^{**}p<0.05$$, $$^{***}p<0.01$$

### Efficiency of online European basketball betting markets

In this section we address the research questions **2a** and **2b** posed in the introduction. In particular, we show the results on the tests for the efficiency (unbiasedness) of online betting markets for the ten major European basketball leagues before and after the closure of the arenas due to the Covid-19 outbreak.

As mentioned in Section 2.2, if betting markets are efficient then the conditional expectation of the bookmaker’s forecast errors should be equal to minus the average margin. Therefore, by estimating the Mincer-Zarnowitz-based model in Eq. (3) and its extensions, we measure that the average margin charged by the bookmakers, ${\widehat{\alpha }}$, and check whether the null hypothesis $H_0 : \beta = 0$ is rejected in favour of the alternative hypothesis $H_1 : \beta > 0$.

The results are reported in Table 5 for the mean odds on the betting market. In Table 9 in the Appendix, we report the results considering the mean normalised odds achieved according to Eq. (2).

**Table 5:** Efficiency of the betting markets

	Bookmaker’s forecast error $\epsilon$		
	${\widehat{\alpha }}$	${\widehat{\beta }}$	*N*
Total sample	–0.0338***	0.0096	27,691
	(0.0067)	(0.0081)	
GG	–0.0288	–0.0135	1027
	(0.0328)	(0.0423)	
Spain	–0.0190	–0.0106	3927
	(0.0201)	(0.0250)	
Russia	–0.0577**	0.0279	1947
	(0.0207)	(0.0240)	
Turkey	–0.0429**	0.0270	3002
	(0.0183)	(0.0227)	
France	–0.0408	–0.0092	3488
	(0.0285)	(0.0378)	
Italy	0.0054	–0.0418	3283
	(0.0273)	(0.0362)	
Germany	–0.0265	–0.0048	3858
	(0.0180)	(0.0220)	
Greece	–0.0448***	0.0537***	2264
	(0.0161)	(0.0184)	
Adriatic	–0.0112	0.0121	2054
	(0.0265)	(0.0325)	
Israel	0.0212	–0.1066***	2182
	(0.0295)	(0.0396)	
Lithuania	–0.0248	–0.0109	1686
	(0.0213)	(0.0246)	

WLS regressions. Estimates of the models in Eq. (3) for the mean odds offered on the betting market. Standard errors in parentheses. $$^*p<0.1$$, $$^{**} p<0.05$$, $$^{***} p<0.01$$

The results in Table 5 show that, considering the mean of the odds proposed by the 47 online bookmakers in our sample, we do not reject the null hypothesis of market efficiency for the leagues analysed. This is also the case for the ghost games played across all the leagues (column “GG” in Table 5). The only exception is the Greek league (HEBA) where the null hypothesis of market efficiency is rejected at 1% significance level. Therefore, HEBA league is the only case where we find market inefficiency that is consistent with the well-known favourite-longshot bias, i.e. betting on favourites provides positive returns. In fact, positive slopes (${\widehat{\beta }}_i>0$) imply that, on average, the bookmaker’s forecast error tends to increase as their forecast implied probabilities increase, i.e. the offered odds decrease.

Moreover, we find that, except for Italian and Israeli leagues, the estimated constant ${\widehat{\alpha }}$, is lower than zero, and in the cases of the whole sample, Russia, Turkey and Greece significantly so. These results imply that the average bookmaker’s margin is around 3.4% in our whole sample. At the individual level, bookmaker’s average margin varies from 1.12% (Adriatic league) to 5.77% (Russia). If, from the one hand, a positive value of ${\widehat{\alpha }}$ is difficult to interpret, from the other hand, it must be noted that, however, all the cases where we find a “positive average margin”, the estimates are not significantly different from zero.

We now consider possible market inefficiencies due to ghost games. The results of the model (4) are reported in Table 6. These results show that betting markets are unbiased (efficient) as no regressor is found significant in all model specifications (1)-(6). Therefore, there is no evidence of an impact of ghost games on the bookmaker’s forecasts, not even in the case of the first three ghost games played (i.e. variable “First_GG” in Table 6). Moreover, as expected, we note that the estimated average margin captured by ${\widehat{\alpha }}$ is always significantly negative at 1% significance level.

**Table 6:** Effect of ghost games on the bookmaker’s forecast error

	Bookmaker’s forecast error $\epsilon$					
	(1)	(2)	(3)	(4)	(5)	(6)
${\widehat{\alpha }}$	–0.0338***	–0.0332***	–0.0331***	–0.0334***	–0.0328***	–0.0332***
	(0.0067)	(0.0067)	(0.0068)	(0.0067)	(0.0067)	(0.0068)
${\widehat{\beta }}$	0.0096	0.0092	0.0091	0.0095	0.0089	0.0092
	(0.0082)	(0.0082)	(0.0082)	(0.0082)	(0.0082)	(0.0082)
GG		–0.0122	–0.0145	0.0058		0.0186
		(0.0118)	(0.0318)	(0.0164)		(0.0443)
FirstGG			0.0033			–0.0190
			(0.0424)			(0.0610)
GG $\cdot p_i$				–0.0364	–0.0546	–0.0729
				(0.0231)	(0.0451)	(0.0628)
FirstGG $\cdot p_i$					0.0335	0.0523
					(0.0586)	(0.0842)

WLS regressions. Estimates of the model (4) when we consider the mean of the odds offered on the betting market. Standard errors in parentheses.

* p$$<0.1$$, ** p$$<0.05$$, *** p$$<0.01$$

Table 9 in the Appendix reports the results for the mean normalized odds. This further step is done in order to rule commissions out of the analysis. This way, the commission is spread equally between the home and away team’s odds. This assumption is actually rather strong, as there is no evidence that the bookmakers symmetrically apply their margin to all the odds. Nevertheless, using the normalized odds is interesting as we note that the favourite-longshot bias is more evident in this case. Moreover, the results from the regressions in Eq. (4) estimated using the normalized odds are summarised in Table 9 in the Appendix. From these results, a favourite-longshot bias is evident, as the impact of the implied probabilities is always significant and positive, thus increasing the bookmaker’s forecast error as the implied probability increases (odds decrease). The impact of ghost games remains non-significant.

We now evaluate the degree of market unbiasedness and whether any biases are large enough to provide profitable opportunities for bettors, which in turn would imply market inefficiency (in line with Angelini and De Angelis (2019) for online European betting markets).

**Fig. 3:**
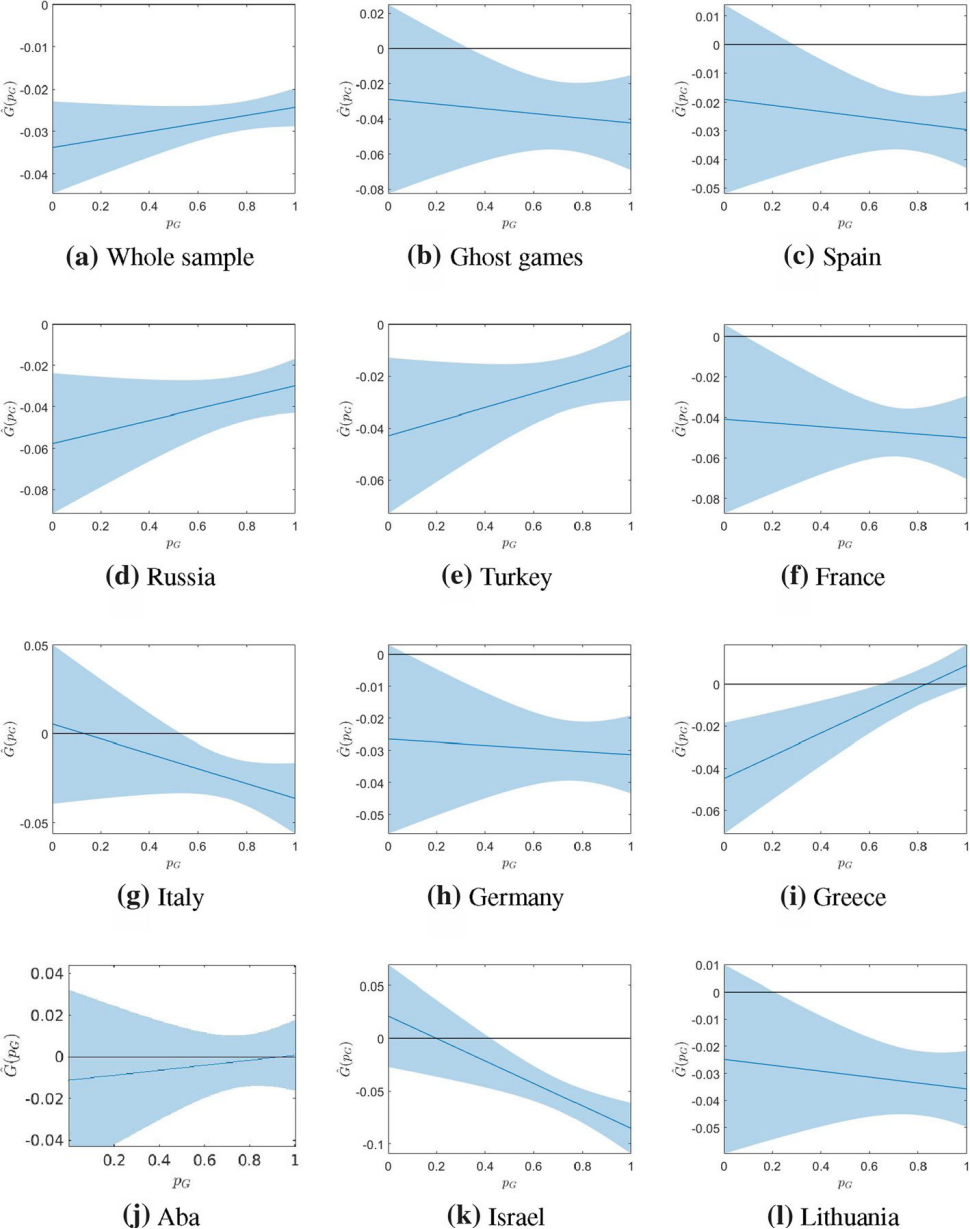
Efficiency curves ${\widehat{G}}(p_g)$ in (5) and related 95% confidence bands in (3.2) computed considering the mean of the odds offered by the betting market

Fig. 3 plots the efficiency curves ${\widehat{G}}$ in Eq. (5) for each league and for the whole sample and ghost games sample, against $p_G \in (0, 1)$ for the mean odds. Fig. 3 shows that all efficiency curves are below the zero line, except for very high values of $p_G$ in the Greek league, or very low values of $p_G$ for the Italian and Israeli leagues. However, the relative 95% confidence bands show that there are no significantly positive values of ${\widehat{G}}(p_G)$. From this empirical evidence we can establish from our estimates that it is not possible for bettors to systematically achieve positive returns. Conversely, significant negative values of ${\widehat{G}}(p_G)$ can be observed for all the cases depicted in Fig. 3, implying that bookmakers are making substantial profits from European basketball betting markets. Moreover, it is interesting to note that the well-documented favourite-longshot bias is not present in all the markets. Indeed, the estimated slope is negative for six leagues and for ghost games (but not if we consider the whole sample), implying a reverse bias, i.e. bookmakers appear to profit more from favourites than from underdogs.^9^

### Determinants of bookmakers’ odds

In this section, we carry out a further analysis to see how the odds offered by the bookmakers have adapted following the introduction of the ghost games.

In particular, we aim at investigating biases in implied probabilities and whether these have adapted, promptly or after a while, to the impact of ghost games on the home advantage. To do this, we estimate using OLS the model in (6) along with some nested alternatives and report the results from such estimations in Table 7.

**Table 7:** Effects of ghost games on the implied probabilities offered by the different bookmakers

	Bookmaker’s implied probability $p_i$					
	(1)	(2)	(3)	(4)	(5)	(6)
GG	–0.0357***	–0.0332***	–0.0380***	–0.0373***	–0.0529***	–0.0519***
	(0.0078)	(0.0078)	(0.0041)	(0.0041)	(0.0126)	(0.0126)
Playoffs		0.0332***		0.0088***		0.0088***
		(0.0046)		(0.0022)		(0.0022)
WElo			0.9752***	0.9748***	0.9753***	0.9749***
			(0.0029)	(0.0029)	(0.0029)	(0.0029)
Matchday					0.0079	0.0078
					(0.0053)	(0.0053)
Matchday$^2$					–0.0008	–0.0008
					(0.0005)	(0.0005)
const	0.6399***	0.6370***	0.1560***	0.1554***	0.1560***	0.1554***
	(0.0014)	(0.0015)	(0.0017)	(0.0017)	(0.0017)	(0.0017)
Adj. $R^2$	0.000811	0.002377	0.771575	0.771679	0.771579	0.771683
F-test (*p*-value)	$<0.0001$	$<0.0001$	$<0.0001$	$<0.0001$	$<0.0001$	$<0.0001$

The dependent variable in all columns is the average implied probabilities offered by the different bookmakers. The model is estimated using OLS. Heteroskedasticity-robust standard errors (HC3) in parentheses.

* $$p<0.1$$, ** $$p<0.05$$, *** $$p<0.01$$

The results reported in Table 7 show that the implied probabilities $p_i$ of the home win odds are significantly affected by ghost games at the 1% significance level. This result suggests that the bookmakers have incorporated into their prices the expected decrease in the home advantage due to the absence of fans in the arena. Such decrease in home advantage is estimated to be around 3.5% for model specifications (1)-(4) and around 5.2% for specifications (5) and (6), which include also the quadratic effect of the number of ghost games played by the home team. Interestingly, we do not find evidence that the adjustment of the bookmaker’s odds is related to the number of matches played behind closed doors as both the linear and the quadratic effects of Matchday are not significantly different from zero. Therefore, the bookmakers immediately and promptly adjusted their odds to take into account the reduction of home advantage in basketball ghost games. The fact that the ghost games variable is a significant determinant of implied probabilities leads us to conclude that online basketball betting markets are (semi-strong) efficient, as information about the decrease in the probability of home winning is incorporated into the odds offered by bookmakers. This result is in contrast with what is found in soccer where such adjustment did not happen at first, hence creating mispricing on the market, as found by Fischer and Haucap (2020) for the German soccer league.

Note that in model specifications (1) and (2) in Table 7, the estimated constant represents the average home win probability predicted by the bookmakers, a result consistent with our analysis of home advantage in Table 2. Indeed, the probability of winning at home in our sample is 61.1%, while bookmakers predict on average about 64%, a percentage that however also includes the margin which we find to be on average about 3.4%.^10^ The results in Table 7 also stress that the WElo rating system is indeed a decent method to measure the home team’s strength. The results from model specifications (3)-(6) show that the estimated coefficient for the WElo variable is close to 1, i.e. the information set provided by the WElo ratings covers almost all of the information set used by bookmakers in setting their odds. However, it must be noted that the null hypothesis of optimal forecast, i.e. $H_0: \delta _3=1$, is strongly rejected (results in specification (6) leads to a *t*-test statistic of $-8.75$), thus highlighting that additional information is used by the bookmakers in the odds-setting process. This evidence can be also inferred from the significant value of the constants in specifications (3)-(6), as for the property of forecast optimality one would expect not to reject the null $H_0: \delta _0=0$, i.e. no bias can be observed when regressing the prediction (implied probability) on the (proxy for the) information set used to achieve such prediction.

## Conclusions

In this paper we investigate the impact of ghost game in indoor sports, with a special focus on the reduction of home advantage due to the absence of supporters inside the arena. We find empirical evidence of a significant reduction of around 5% of the home winning probability in the top ten European basketball leagues in 2020, i.e. when the basketball leagues resumed playing behind closed doors. Moreover, this reduction does not seem to disappear over time, suggesting that familiarity with home surrounds is not a factor in explaining the home advantage. These results are in line with previous findings with regard sporting events carried out in the wake of the Covid-19 Pandemic.

Finally, we find substantial differences in the reaction of the online betting markets for basketball and soccer. In particular, results in the recent literature show that bookmakers only solved the bias due to ghost games in soccer betting markets through a weak adaptation process over time, whereas in basketball the bookmakers appear to have foreseen the home advantage reduction in advance, perhaps due to the fact that basketball resumed later than soccer did after the first wave of the Covid-19 Pandemic in the Spring of 2020, thus avoiding to create biases and inefficiencies in the market. This evidence could also be related to our main conjecture, that indoor sports, and basketball in particular, are more inclined to be affected by closed door games—and permanently so—than outdoor sports like soccer.

Future research could investigate whether similar evidence can be found for other indoor sports such as volleyball or ice hockey. Moreover, it would be interesting to relate our findings on European basketball to the findings on basketball in the US.

## Appendix

**Table 8:** Details on the top 10 European leagues

League	Country	# of teams	Home games	# of teams	Finals	Capacity (2018-19)			Attendance (2018-19)		Season 2019-20
		(2020-21)	(regular season)	in playoffs	(best of)	Min	Max	Average	Max	Average	
ACB Liga	Spain	18	17	8	5	5,000	15,504	8,719	15,544	6,236	Started on 2019/09/24, suspended on 2020/04/02, ended on 2020/06/30 with a closed door tournament in Valencia
VTB United League	Russia	13	12	8	5	4,000	15,000	7,643	7,389$^\dagger$	2,343$^\dagger$	Started on 2019/09/25, suspended on 2020/03/12
Basketbol Süper Ligi	Turkey	16	15	8	5	1,250	16,000	7,008	–	–	Started on 2019/09/28, suspended on 2020/03/18
LNB Pro A	France	18	17	8	5	2,000	7,707	4,631	–	–	Started on 2019/09/21, suspended on 2020/03/27
Lega Basket Serie A	Italy	16	15	8	7	3,506	12,700	6,172	12,005	4,003	Started on 2019/10/25, suspended on 2020/03/08
Basketball Bundesliga	Germany	18	17	8	5	3,000	14,500	5,172	–	4,189	Started on 2019/09/24, suspended on 2020/03/25, ended on 2020/06/30 with a closed door tournament in Munich
HEBA Basket League	Greece	12	11	8	5	1,204	19,250	5,902	–	–	Started on 2019/09/28, suspended on 2020/03/08
ABA Liga	Adriatic$^*$	14	13	4	5	2,500	12,480	4,992	8,000	2,691	Started on 2019/10/04, suspended on 2020/03/09
Winner League	Israel	13	14/15	8	3	1,200	11,000	3,906	–	–	Started on 2019/10/05, suspended on 2020/03/13, ended on 2020/07/28 with a closed door tournament in Tel Aviv
LKL	Lithuania	10	18	8	5	1,500	15,708	5,342	11,294	2,478$^\dagger$	Started on 2019/10/04, suspended on 2020/03/13

$^*$Bosnia and Herzegovina, Croatia, North Macedonia, Montenegro, Serbia, and Slovenia

$^\dagger$Data related to 2017-18 season

Here we report the results for the test for efficiency of betting markets using normalised odds according to Eq. (2) in Table 9. The favourite-longshot bias is more evident than the case of mean odds, as the slope ${\widehat{\beta }}>0$ for all the leagues and significant for four leagues out of ten as well as for the total sample − thus the bookmaker’s forecast error tends to increase as the implied forecast probability increases. The only exception is Israeli league where we observe a reverse bias. It is worth noting that the normalisation procedure adopted cannot fully set to zero $\alpha$, i.e. the parameter that captures the bookmaker’s margin, as it is found significantly different from zero for Russia, Greece as well as for the whole sample. Quite surprisingly, ${\widehat{\alpha }}$ for Turkey is found significantly positive. As in the case of mean odds, the impact of ghost games is still insignificant (see row “GG” in the table).

**Table 9:** Efficiency of betting markets (normalised odds)

	Bookmaker’s forecast error $\epsilon$		
	${\widehat{\alpha }}$	${\widehat{\beta }}$	*N*
Total sample	–0.0261***	0.0571***	27,691
	(0.0063)	(0.0093)	
GG	–0.0199	0.0270	1027
	(0.0313)	(0.0482)	
Spain	–0.0109	0.0316	3927
	(0.0193)	(0.0277)	
Russia	–0.0508***	0.0772***	1947
	(0.0178)	(0.0257)	
Turkey	0.0442***	0.0954***	3002
	(0.0172)	(0.0255)	
France	–0.0215	0.0179	3488
	(0.0284)	(0.0420)	
Italy	0.0142	0.0002	3283
	(0.0274)	(0.0407)	
Germany	–0.0148	0.0317	3858
	(0.0171)	(0.0248)	
Greece	–0.0466***	0.1252***	2264
	(0.0150)	(0.0216)	
Aba	–0.0013	0.0615*	2054
	(0.0261)	(0.0376)	
Israel	0.0434	–0.0894**	2182
	(0.0291)	(0.0452)	
Lithuania	–0.0264	0.0670***	1686
	(0.0174)	(0.0259)	

WLS regressions. Estimates of the models in (3) for the normalised odds offered on the betting market. Standard errors in parentheses. $$^{*} p<0.1$$, $$^{**} p<0.05$$, $$^{***} p<0.01$$

## Footnotes

1 Other factors may affect home advantage, such as the distance travelled by the away team, the familiarity of the home ground or of the tactical orientation.

2 The top ten leagues are defined according to: https://www.eurohoops.net/en/league-action/938659/domestic-leagues-top10-spain-vtb-and-the-others/

3 In the sake of space, the results from the estimation of the logit and probit models are not reported and are available upon request.

4 The implied probability can be obtained as the reciprocal of the odds offered by the bookmaker.

5 Here European countries mean states that are members of the European Basketball Federation.

6 Note that matches played with a very low percentage of attendance due to Covid-19 regulations are also considered as ghost games. Although this may seem unsatisfactory, as Reade et al. (2021) provide graphical evidence suggesting that this distinction matters for soccer, the albeit small-sample evidence is that in basketball, the distinction doesn’t matter so much. Our dataset has 868 ghost games, of which 72 are played with a restricted number of fans. In the games without any fans the win percentage is 58%, and in the games with restricted numbers, the win percentage is 54%.

7 The complete list of the online bookmakers can be found on the oddsportal website and available upon request.

8 The results for the logit and probit models are very similar to those achieved using the linear probability model. Therefore, despite the latter being the less appropriate statistical model, we have decided to show the results from the OLS linear model because its interpretation is straightforward.

9 Conversely, considering the normalised odds, we find evidence of inefficiencies and profitable opportunities for bettors in these markets due to the elimination of the bookmaker’s margin. Obviously, as we cannot find normalized odds in real life, these empirical evidences are only theoretical.

10 Considering the regressions made using the normalized odds, i.e. where the margin is excluded, the results are fully consistent with the home win probabilities reported in Table 2.
